# Engineered Nanoparticle-Protein Interactions Influence Protein Structural Integrity and Biological Significance

**DOI:** 10.3390/nano12071214

**Published:** 2022-04-05

**Authors:** Surabhi Jaiswal, Amit Manhas, Alok Kumar Pandey, Smriti Priya, Sandeep K. Sharma

**Affiliations:** 1 Biomolecular Toxicology Lab, Food, Drug and Chemical Toxicology Division, CSIR—Indian Institute of Toxicology Research, Lucknow 226001, India; surabhijaiswal.11@gmail.com (S.J.); amitashu.manhas@gmail.com (A.M.); 2 Academy of Scientific and Innovative Research (AcSIR), Ghaziabad 201002, India; 3 System Toxicology & Health Risk Assessment Group, CSIR—Indian Institute of Toxicology Research, Lucknow 226001, India; alokpandey@iitr.res.in (A.K.P.); spriya@iitr.res.in (S.P.)

**Keywords:** engineered nanoparticles, protein, protein folding, misfolding, aggregation, protein corona, molecular chaperones, nanochaperones

## Abstract

Engineered nanoparticles (ENPs) are artificially synthesized particles with unique physicochemical properties. ENPs are being extensively used in several consumer items, elevating the probability of ENP exposure to biological systems. ENPs interact with various biomolecules like lipids, proteins, nucleic acids, where proteins are most susceptible. The ENP-protein interactions are mostly studied for corona formation and its effect on the bio-reactivity of ENPs, however, an in-depth understanding of subsequent interactive effects on proteins, such as alterations in their structure, conformation, free energy, and folding is still required. The present review focuses on ENP-protein interactions and the subsequent effects on protein structure and function followed by the therapeutic potential of ENPs for protein misfolding diseases.

## 1. Introduction

Engineered nanoparticles (ENPs) are artificially synthesized particles that are nano-scaled (1–100 nm) in at least one dimension and have distinctive properties from their bulk counterparts [1,2]. ENPs can be synthesized using two different approaches, namely top-down and bottom-up approaches (Table 1) [1,3]. In the top-down approach, nanoparticles are produced by mechanical breakdown of bulk material using methods such as lithography, mechanical milling, electrospinning, laser ablation etc, that provides advantage of producing ENPs of controlled size and morphology [4]. In the bottom-up approach, molecules are assembled to form ENPs by sol-gel method, chemical vapour deposition (CVD), reverse micelle methods etc.

**Table 1:** Techniques used in the synthesis of nanoparticle.

Nanoparticle Synthesis Approach	Techniques	Description	Reference
Top-down approach		Synthesis is initiated by systematic leaching of bulk counterpart leading to the generation of nano-scaled particles.	
	Lithography	Categorized as masked and maskless lithography. In masked lithography transfer of nano-patterns over a large surface area is done using a specific mask e.g., photolithography and soft lithography. In maskless lithography, arbitrary nanopattern is written without using any mask e.g., electron beam lithography and focussed ion beam lithography.	[5]
	Mechanical milling	Formation of nano-scaled material by elastic, plastic and shear deformation followed by fracture, amorphization and chemical reactions. It helps in nanocomposite production.	[6]
	Electrospinning	Used for nanofibres production from various materials, typically polymers.	[7]
	Sputtering	Nanomaterials are produced by the bombardment of high-energy particles such as gas or plasma on the solid surface. Used for the production of thin films of nanomaterials.	[8]
	Arc discharge method	Used for the production of carbon-based materials such as fullerenes, carbon nanotubes etc.	[9]
	Laser ablation	Nanomaterials synthesized using a powerful beam of laser that hits the target.	[10]
Bottom-up approach		Synthesis is by coalescence or assembling of atoms and molecules to produce various nanoparticles.	
	Sol-gel method	Metal oxide nanoparticles are synthesized by the transformation of liquid precursor to a sol followed by its conversion into a gel.	[11]
	Chemical vapour deposition	Nanomaterials are synthesized by thin film formation over the surface of the substrate due to the chemical reaction of vapor-phase precursors.	[12]
	Hydrothermal and solvothermal methods	Nanomaterials are synthesized in either aqueous medium (hydrothermal method) or non-aqueous medium (solvothermal method) by heterogeneous reaction under high pressure and temperature near the critical point in an enclosed vessel.	[13]
	Template methods	These are used to synthesize nanoporous materials either by using a soft template such as block polymers and surfactants or by using a hard template such as carbon nanotubes, carbon black, wood shells, silica and colloidal crystals.	[14,15]
	Reverse micelle methods	Nanoparticles are synthesized by the formation of reverse micelle which is created in the case of water-in-oil emulsion where hydrophilic heads point towards the core. This core act as a nanoreactor for nanoparticle synthesis.	[16]

Based on their composition, ENPs can be categorized into organic, inorganic, and hydrogel nanoparticles (Table 2). Because of the unique physicochemical properties such as size, surface to volume ratio, zeta potential, optical, magnetic and catalytic properties, ENPs are being extensively used in numerous consumer and industrial products such as healthcare products, food, cosmetics, packaging, paints, sensors, and electronic devices. The global market value of nanomaterials was approximately 14.7 billion US Dollars in 2015 and it is likely to reach 55 billion USD by 2022, at over 20.7% of compound annual growth rate (CAGR) during the forecast duration 2017–2022 [17]. The wide applications of ENPs have raised concerns regarding their safety on the environment and living organisms. ENPs from various sources such as industries, automobile exhaust, consumer products enter the ecosystem through water bodies, air, and soil. Owing to their accumulation ability, ENPs undergo trophic transfer through terrestrial and aquatic food chains [18,19,20]. Therefore, humans are exposed to ENPs through the use of various consumer items and the ecosystem contaminated with ENPs. ENPs can interact with biomolecules like nucleic acids, lipids and proteins once they enter inside a biological system making “biomolecule corona”. Since proteins regulate metabolic pathways and cellular homeostasis, it is imperative to understand the interaction of ENPs with proteins. 

**Table 2:** Types of ENPs, their characteristics, and applications.

ENPs Types	Size (nm)	Characteristics	Applications	References
Organic ENPs				
Dendrimers	<10 nm	Radially symmetric molecules with highly branched structures made of one or more cores. These are homogeneous and monodispersed.	Controlled and targeted bioactive delivery to macrophages, liver targeting, transdermal drug delivery, gene delivery	[21,22,23]
Liposome	50–100 nm	Vesicles of phospholipid with superior entrapment ability. These are biocompatible and versatile.	Passive and active gene delivery, can be used for peptides, proteins, and cell interactions studies, anti-cancer therapy	[24]
Polymeric ENPs	10–1000 nm	Biodegradable and biocompatible.	Controlled and sustained drug delivery carriers, protein carriers, intra-arterial localization of therapeutic agents	[25]
Micelles	10–100 nm	Formed of amphiphilic molecules like polymers and lipids.	Targeted delivery of siRNA and anticancer drug, diagnosis	[26,27]
Inorganic ENPs				
Metallic	<100 nm	Metal colloids with a high surface-to-volume ratio. These are stable and have better mechanical strength, optical and magnetic properties.	Delivery of genes and drugs, ultrasensitive diagnostic assays, radiotherapy, and thermal ablation	[28,29,30,31]
Metal oxide	<100 nm	Oxides of metals with antioxidant activities, chemical stability, catalytic and optical properties, and biocompatibility.	Medical implants, drug delivery, biological antioxidant, bioimaging, biosensors	[32,33,34]
Ceramic	<50 nm	Non-metallic solids of non-metallic and metallic compounds with the property of heat resistance.	Bone repair, drug delivery vehicles, photocatalysis, imaging, photodegradation of dyes	[35]
Nanocrystal Quantum dots	2–9.5 nm	Semiconductive material consists of a semiconductor core, a shell, and a cap. These have high photostability, broad UV excitation, narrow emission, bright fluorescence, and resistance to photobleaching.	Long-term multi-colour imaging of hepatocytes, DNA hybridization, immunoassays, receptor-mediated endocytosis, disease marker labeling	[36,37,38]
Fullerenes	1–2 nm	High strength, electrical conductivity, electron affinity, and versatile structure.	Gene and drug delivery, antiviral activity	[39,40]
Carbon nanotubes	0.5–3 nm in diameter, 20–1000 nm in length	These are single or multi-walled nanotubes with unique strength and electrical properties. Found in crystalline form.	Can penetrate inside cell and nucleus, gene and peptide carrier, used in imaging, drug delivery, tissue engineering	[41]
Hybrid ENPs				
Hydrogels	0.1 to 100 μm	These are also known as polymeric nanogels and macromolecules micelles. These are polymeric networks having a three-dimensional structure with high water or biological fluid absorbing capacity owing to the hydrophilic groups present in the polymer chains.	These are used in the delivery of drugs of small molecular weight, peptides, proteins, nucleic acids, oligosaccharides and vaccines.	[42,43]

To become functional, the polypeptides must fold into a three-dimensional structure termed as the native form of the protein, that may exist as a monomer, dimer, or polymer. During protein folding, nascent polypeptides form intermediate structures that are highly unstable due to their high entropy and free energy [44]. These intermediates either fold into a native structure or may get misfolded. Misfolded or partially folded proteins tend to form stable protein aggregates that may lead to various misfolding diseases such as Huntington’s disease (HD), Alzheimer’s disease (AD), Parkinson’s disease (PD) and Creutzfeldt-Jacob disease (CJD). Molecular chaperones, also known as heat shock proteins (HSPs) play an important role in maintaining protein homeostasis, preventing protein misfolding and refolding of misfolded and aggregated proteins [45]. Proteins have a dynamic nature, therefore, any type of change in their environment such as pH, temperature, crowding, free energy may alter the protein conformation, structure and function. The presence of ENPs in the protein environment may alter proteins either by adsorption of proteins over ENPs surface forming a protein corona or by influencing the protein folding process. The protein corona formation can change the biological identity of ENPs by surface modification, alteration of zeta potential, size, reactivity, catalytic and magnetic properties and can cause alterations in the protein conformation, stability, functionality, aggregation and kinetics. ENPs can also affect protein conformation and function by mimicking molecular chaperones and therefore, can supplement existing therapeutic strategies for protein folding diseases. Therefore, apart from studying changes in the properties of ENPs as a result of protein corona formation, understanding of protein modulation ability of ENPs is very important and still needs further studies. This review focuses on the alterations in proteins due to ENP-protein interaction and possible therapeutic applications of ENPs for protein misfolding diseases.

## 2. ENPs Interaction with Proteins

Internalization of ENPs inside the biological system is followed by their interaction with proteins present in the extracellular matrix such as elastin [46], histidine-rich glycoprotein [47], cytoskeletal proteins such as vimentin, lamin B1 and gelsolin [48], proteins bound to membranes such as nicotinamide adenine dinucleotide phosphate (NADPH)-oxidase (NOX) [49] and integrins, cytosolic and organellar proteins such as superoxide dismutase (SOD), catalase [50] and nucleoplasmic proteins such as topoisomerase I [51]. This means ENPs can interact with a wide range of proteins involved in essential cellular events such as cell cycle regulation, proliferation, transcription, signal transduction, cellular metabolism and apoptosis. Since these processes are majorly dependent on the proper functioning of proteins which is directly linked to protein folding, therefore, ENPs interaction with any of the states of protein formed the during protein folding process can play a major role in the alteration of biological processes. The alterations in the protein can be either due to the formation of protein corona or due to the presence of ENPs in the protein proximity.

### 2.1. Protein Corona

Proteins are among the first molecules which interact with ENPs when ENPs enter the biological system. The adsorption of proteins over the surface of ENPs results in the formation of a corona-like structure which is known as protein corona. At any particular time, plasma proteins, kinetics and the equilibrium binding constants of protein for a specific ENP determines the composition of protein corona [52]. Initially, highly abundant plasma proteins with high association rates form the layer over ENPs, that are subsequently replaced by proteins having higher affinity and longer time of residence. Based on adsorption strength and residence time of proteins on the ENP surface, two types of protein corona exist: one is hard corona with strong adsorption ability and long residence time of proteins over the ENPs surface and the other is soft corona which is having a shorter residence time and lower affinity for adsorption of proteins onto ENPs [52,53,54,55,56]. Protein corona has been observed on various ENPs such as polymeric ENPs [57,58], quantum dots [59], iron oxide ENPs [60,61], AgNPs and AuNPs [62,63]. Protein corona changes the biological identity of ENPs and therefore bio-reactivity of ENPs [64]. Protein corona determines cellular uptake, inflammatory responses, accumulation, degradation and removal of ENPs [65]. Several transport proteins such as apolipoprotein E forms portein corona with ENPs, that plays a crucial role in trafficking in the brain and can be helpful in the development of neurotherapies [52,66,67]. On the other hand, ENPs have been found to cause structural and conformational changes in the adsorbed proteins which alter the normal functioning of proteins [68,69]. Structural changes in proteins after adsorption over ENPs surface have biological significance as loss-of-function or gain-of-function are associated with the generation of unwanted immune responses and maintenance of physiological homeostasis [69]. Secondary structure disruption of insulin along with the induction of aggregation by quantum dots based on their specific size and shape shows the proteopathy inducing potential of ENPs [70]. Therefore, the formation of protein corona is a critical parameter of ENPs need to be considered for applications in nanomedicine and nanocarriers. 

### 2.2. Role of ENPs in Protein Folding Pathway

In the protein folding pathway, starting from the formation of nascent polypeptide chains on the ribosome till the formation of the native protein, several intermediates are formed. Disruption of the protein folding process due to external or internal stresses may result in protein misfolding. Owing to the ability of protein interaction, ENPs can severely affect the process of protein folding [71,72], aggregation [73,74] and fibrillation [75,76]. This characteristic of ENPs has been studied extensively due to the potential effect of ENPs on various essential proteins. 

#### 2.2.1. Role of ENPs in Protein Unfolding

Stress conditions such as changes in pH, temperature, or any other external and internal stimulus can result in the unfolding and denaturation of proteins. Protein unfolding is a very crucial step in deciding the fate of partially folded or misfolded proteins that is whether they go for correct refolding or proceed towards the formation of aggregates and fibrils. ENPs assist in unfolding by denaturation of protein over the surface of nanoparticles [77]. This provides misfolded protein conformers an opportunity to refold to their native form.

#### 2.2.2. Role of ENPs in Protein Folding

Folding of the nascent polypeptide or refolding of unfolded protein to acquire its functional native form is crucial for maintaining protein homeostasis and avoiding diseases associated with protein misfolding. ENPs such as zinc oxide nanoparticles (ZnONP), alumina nanoparticles and nanoliposomes can assist in protein refolding [78,79,80]. Nanoliposomes can attach to misfolded amyloid light chain protein which is associated with the pathogenesis of a protein-misfolding disease named light chain amyloidosis (AL) and reduce endothelial tissue injury, therefore, such ENPs can play a significant role in the treatment of protein misfolding diseases [80]. However, some ENPs can also inhibit enzyme activity and alter secondary structures which indicate towards ENPs mediated formation of misfolded conformers [81].

#### 2.2.3. Role of ENPs in Protein Aggregation

Misfolding of proteins is a deleterious process if it proceeds towards the formation of protein aggregates and fibrils. Prevention and inhibition of aggregate formation could provide a possible way to deal with protein misfolding therefore, ENPs have been investigated for their inhibitory effect on protein aggregates. ENPs can inhibit amyloid β (1–42) peptide aggregation and substantially disintegrate pre-formed aggregates, stabilize α-helix, inhibit the β-sheet formation and alleviate related cytotoxicity [82,83]. However, ENPs can also accelerate protein aggregation by influencing the nucleation and growth phase [84].

#### 2.2.4. Role of ENPs in Protein Fibrillation

**Figure 1:**
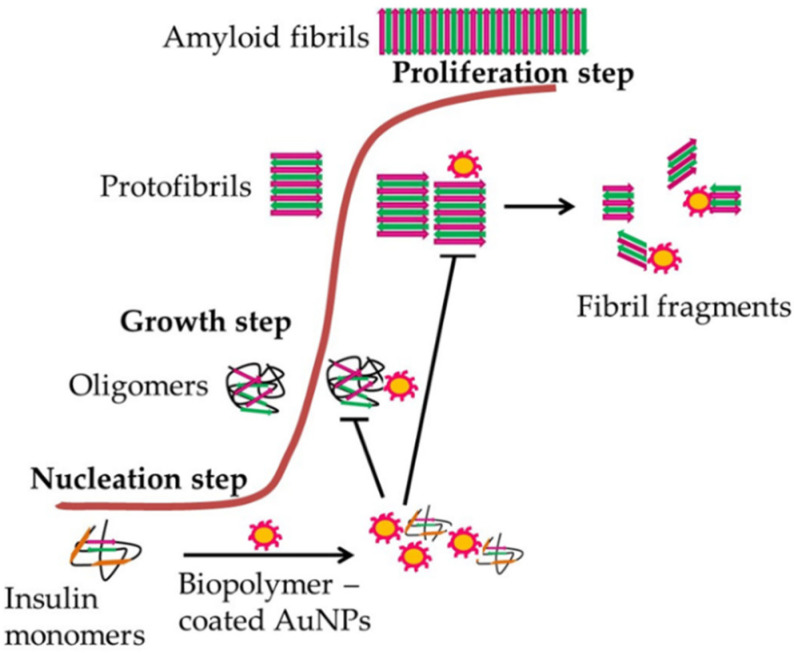
Interaction mechanism of biopolymer-coated AuNPs mediated inhibition of insulin amyloid fibrillation. The formation of amyloid fibrils is a multi-step process. Interaction of biopolymer-coated AuNPs with insulin monomers during the nucleation step of fibrillation inhibits the insulin amyloid fibril formation. These ENPs strongly interact with insulin monomers via their –OH and –NH_2_ groups thereby, inhibiting oligomer formation and protofibril elongation resulting in the formation of thin and short fibrils.

The internalization of ENPs in the biological systems can cause a series of complicated biological reactions, one of which can be promotion or inhibition of protein fibrillation which is associated with several misfolding diseases such as AD and PD [81]. ENPs like AuNPs of different shapes have been found to catalyze the nucleation step of fibrillation by interfacial adsorption of amyloid-β (1–40) [81]. While another study performed using biopolymer-coated AuNPs showed that ENPs can inhibit insulin fibril formation by strong interaction between biopolymer-coated AuNPs and protein monomers in the nucleation step of fibril formation causing inhibition of oligomer and protofibrils formation (Figure 1) [75].

## 3. Factors Responsible for ENP-Protein Interactions

**Figure 2:**
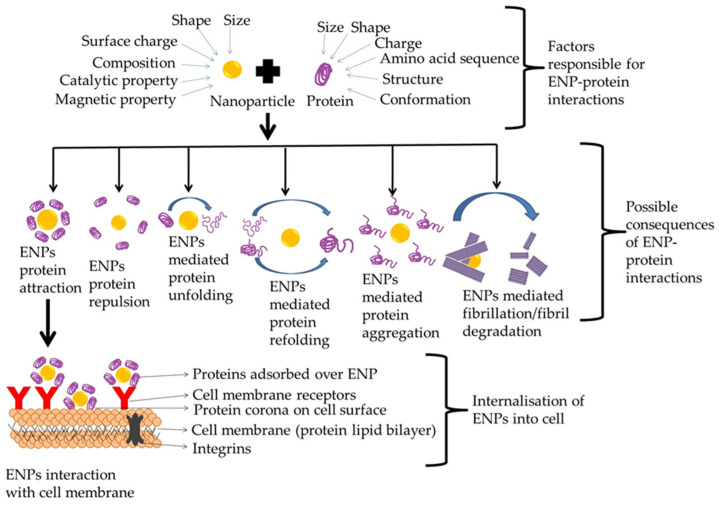
Factors governing ENP-protein interactions and the subsequent effect of these interactions on the protein conformation and function. ENP-protein interaction results in attraction between ENPs and proteins having opposite charges and repulsion between like charges. As proteins are among the first molecules with which ENPs interact after their entry inside the biological systems, therefore, this interaction facilitates passage through the semi-permeable plasma membrane of the cells. Further, these ENPs may modulate protein folding processes such as unfolding, refolding, misfolding, aggregation and fibrillation.

ENP-protein interactions are majorly dependent on two aspects: first is the interaction forces that is the non-covalent interactions occurring between nanoparticle surface and protein surface and second is the ratio between an ENPs size and a protein size [85]. Factors affecting the ENP-protein interactions include size, zeta potential, surface area, radius of curvature, surface functionalization, coating, hydrophobicity and hydrophilicity (Figure 2) [85,86,87,88,89,90].

### 3.1. Size and Radius of Curvature

The size of ENPs and proteins is an important factor that decides the mode of interaction as ultra-small ENPs (about 1–2 nm) interact with the binding region or specific epitopes present on the surface of larger proteins, medium-sized ENPs (about 5 nm) which are comparable in size with proteins interact with each other like two ENPs or two proteins and large-sized ENPs (above 20 nm) owing to their high radius of curvature act like a two-dimensional surface even for larger proteins, therefore the proteins get adsorbed over ENPs surface [85]. The curvature effect of ENPs on protein adsorption depends on the way that if the size of the nanoparticle increases, the surface to volume ratio decreases which leads to decreased amount of protein adsorption and vice versa.

### 3.2. Surface Charge

The surface charge of ENPs and charge on amino acid residues of the proteins decide the binding affinity and type of interaction such as Van der Waals, electrostatic interactions and covalent interactions. ENPs and proteins having like charges are repelled while those with unlike charges are attracted towards each other. The surface charge-dependent ENP-protein interactions cause changes in protein structure such as inhibition of the fibrillation process due to electrostatic interaction between oppositely charged β-sheet forming protein residues and ENPs surface irrespective of their shape, size and composition [91]. 

### 3.3. Shape

ENPs are available in different shapes such as spherical, pyramidal, cylindrical and plate-shaped. The binding affinity of proteins with ENPs and the thickness of protein layers adsorbed on the surface of ENPs is highly dependent on the shape of ENPs. It has been observed that proteins show three times higher affinity for spherical shaped AuNPs as compared to branched AuNPs of the same size [92]. The shape of ENPs is an important deciding factor for surface-to-volume ratio. The high surface-to-volume ratio of ENPs increases the number of proteins adsorbed per unit surface area of ENPs which enhances the contact of unfolded proteins, causing clustering of proteins at a faster rate or formation of new protein clusters [93].

### 3.4. Affinity and Exposure Time

Adsorption of proteins over ENPs surface depends on the comparative affinity of different proteins and time of exposure. This is explained by the Vroman effect which governs the time-dependent adsorption of proteins over ENPs surface. According to this, proteins having less affinity for ENPs and shorter exposure time are replaced with proteins having a higher affinity for ENPs and longer exposure time. This results in soft and hard corona formation [94]. 

### 3.5. Thermodynamic Parameters

ENP- protein interaction is controlled by thermodynamic parameters such as entropy and free energy. These parameters are interrelated as ∆G = ∆H − T∆S, where ∆G represents a change in Gibbs free energy, ∆H represents a change in enthalpy, T represents absolute temperature and ∆S represents a change in entropy. These reveal the spontaneity of adsorption and binding of proteins with ENPs based on the free energy (ΔG) of the system [95].

### 3.6. Biofluid

The interaction between ENPs and proteins may be of non-covalent, hydrophobic and non-specific type, therefore, there is an important role of solvents in these interactions. With the increment in the biological nature of the media that is from pure water to PBS, protein-free cell culture media to that with protein supplementation and then to actual biological media, binding of proteins to the ENPs differs significantly. This might be a reason why some of the effects of ENPs-protein interactions that have been observed during in vitro studies are not found during in vivo studies [96,97,98].

## 4. Chaperoning Functions of ENPs

The protein folding process occurs in several steps forming intermediate states of protein supported by various chaperones and chaperonins. Molecular chaperones work by interacting with, stabilizing and repairing non-native forms of proteins. In addition to this, they also bind to nascent polypeptides inhibiting undesired intermolecular interactions and protein aggregation. Some ENPs can act as chaperones or chaperonins because of favourable protein orientations on their scaffold which helps in preventing the aggregate formation and refolding of protein into the native form [99,100]. This property of nanoparticles drawn the attention towards the development of ENPs which can mimic molecular chaperones (nanochaperones) [101]. 

### 4.1. ENPs Have Been Categorized as Chaperone-Mimicking ENPs and Chaperone-Aiding ENPs Based on Their Structure and Functions

#### 4.1.1. Chaperone-Mimicking ENPs

These have structural and behavioural similarities with molecular chaperones and are also known as artificial chaperones. These ENPs have been shown to prevent aggregation and assist in refolding of non-amyloidogenic proteins such as RNase and lysozyme [102]. The earliest artificial chaperones were made based on the catch and release mechanism of the GroES/GroES chaperone system found in prokaryotes [103]. The working efficiency of artificial chaperones depends on their binding affinity to different forms of proteins. Polymer hydrogel nanoparticles are one such artificial chaperones that were found to counteract positively charged lysozyme aggregates by strongly interacting with denatured protein and weakly interacting with the native form of protein thereby, facilitating resolubilization of aggregates and refolding. However, this facilitation is not observed in ENPs showing low affinity to the denatured form of protein or high affinity for both native and denatured proteins [102].

#### 4.1.2. Chaperone-Aiding ENPs

These are functionally similar to molecular chaperones and are also known as chemical chaperones. They assist protein assembly, stabilize and assist refolding of unfolded proteins and mediate protein degradation [104]. These ENPs show therapeutic potential for conformational disorders by assisting the protein folding process and aggregates clearance of proteins such as amyloid β and α-synuclein. Hsp-inspired, mixed-shell polymeric micelle (MSPM) based self-assembling nanochaperone is one of the chaperone-aiding ENPs which selectively captures Aβ peptides, suppress the Aβ aggregate formation process and reduce cytotoxicity mediated by Aβ, facilitate Aβ clearance thereby, reducing Aβ burden, attenuate Aβ-induced inflammation and were able to rescue cognitive deficits in APP/PS1 transgenic AD mice indicating the potential application of MSPM based nanochaperones for prophylactic treatment of AD and prevention of the onset of AD-like symptoms [105].

### 4.2. ENPs with Chaperone-like Activity

These ENPs can prove as a promising strategy for effective correction and maintenance of protein homeostasis. These ENPs can assist in protein refolding, sequestering misfolded proteins, inhibiting amyloid β aggregation, clearance of amyloidogenic proteins and fibril degradation. 

#### 4.2.1. ENPs Assisted Refolding of Proteins

This occurs by the interaction of denatured proteins with ENPs. These ENPs follow the protein folding mechanism of natural molecular chaperones or chaperonins such as the GroEL/GroES system which forms a nano-cage for isolated folding of a protein molecule. Polymeric micelles synthesized by self-assembling poly(ethylene glycol)-phosphatidylethanolamine (PEG-PE) show structural similarity with bacterial GroEL/GroES chaperonin system as they have hydrophilic nano-cage with a negatively charged layer. Owing to its structural similarity, PEG-PE micelle can assist in refolding of denatured insulin and avoid protein aggregate formation [106].

#### 4.2.2. ENPs Assisted Modulation of Protein Misfolding

This occurs mainly by altering the nucleation step where ENPs act as destabilizing agents or as microreactors leading to acceleration or retardation of amyloidosis [99,107]. Since amyloid proteins are unstructured as compared to native proteins, therefore, the exposed hydrophobic sequences facilitate fibril formation. The exposed hydrophobic regions increase the sensitivity of protein misfolding pathways for ENP- protein interactions and governing factors. This interaction depends on factors such as protein corona formation and its strength, peptide sequence, physiological conditions (such as pH, ionic strength, temperature) and ENP- peptide ratio. 

#### 4.2.3. ENPs Assisted Clearance of Amyloid Proteins

This is based on the working mechanism of chaperone-mediated autophagy (CMA) which facilitates the removal of amyloidogenic proteins [108]. Amyloidogenic proteins are an important category of proteins that undergo the process of amyloidosis which causes increased β-sheet structures which are directly linked to protein misfolding, aggregation and fibrillation that are associated with various neurodegenerative diseases such as AD and PD. These smart ENPs or nanosweepers consist of two functional units namely the capture unit and the clearance unit. A small peptidic sequence that recognizes Aβ can act as a capture unit and an antibody such as Beclin-1 which plays an important role in autophagy regulation performs the function of a clearance unit [109]. The ENPs made from acrylate-modified chitosan act as nanosweepers. Here, the capture unit is a thiol-functionalized KLVFF peptide, and the clearance unit is a thiol-functionalized Beclin-1 which are linked by acrylate double bond to chitosan through Michael additions [110].

#### 4.2.4. ENPs Assisted Fibril Degradation

This has been proposed as a possible strategy for the treatment of amyloid diseases. For effective ENPs mediated defibrillation of amyloid fibrils, proper selection of core material, modification of ENPs surface and duration of ENPs administration are probable key factors to be considered [111]. Owing to their plasmonic photothermal property, silver triangular nanoplates (AgTNPs) can cause the dissolution of mature Aβ fibrils. Mature Aβ fibrils treatment with AgTNPs resulted in the dissolution of the fibrils in less than an hour under near-infrared (NIR)-illuminated conditions, while spherical shaped silver nanoparticles of the same concentration needed 70 h. In addition to this, by the selective binding of positively charged amyloidogenic sequences present in the Aβ monomer with AgTNPs, Aβ fibrillation was prevented [112].

## 5. Therapeutics Based on ENPs-Protein Interactions

Owing to their nano size, ability to interact with biomolecules, to cross the highly selective blood-brain barrier (BBB), anti-amyloidogenic activity, chaperone mimicking ability, ENPs can provide a possible strategy for the treatment of several diseases such as PD, AD, HD and cancer, etc.

### 5.1. ENPs Controlled Protein-Ligand Binding Efficiency

ENPs can efficiently control the binding of ligands such as curcumin (which are considered anti-cancer agents) with proteins. In this, the size of ENPs plays an important role as observed during the binding of curcumin with lysozyme in presence of three different sizes of silver nanoparticles (AgNPs) which changed the binding affinity of curcumin to lysozyme and cause the varied amount of conformational changes in the protein [113].

### 5.2. Anti-Amyloidogenic Activity of ENPs

Due to their anti-amyloidogenic activity, ENPs can provide promising strategies for the treatment of amyloid involving neurodegenerative disorders. Some ENPs such as dendrimer- tesaglitazar (D-tesaglitazar) conjugate have shown the potential for the treatment of multiple neurological disorders by degradation of pathogenic proteins and β-amyloid clearance [114]. ENPs can interfere with the initiation of the fibril formation process. Amyloid fibril formation occurs in multiple steps and is associated with several protein misfolding diseases. The N-terminus amphipathic “KA/TKE/QGV” repeating motifs of α-synuclein which is an amyloidogenic protein is associated with PD, was found to interact with ZnONP. ZnONP kinetically traps α-synuclein fibrillation by trapping the monomers into the mesh-like amorphous aggregates (flocs) thus creating interference in initial intermolecular interaction between α-synuclein monomers which are needed for the fibrillation process [115]. The neurotransmitter functionalized ultra-small-sized gold nanoparticles (USGNPs, ~4 nm diameter) can effectively inhibit fibrillation and increase cell viability which makes USGNPs a suitable candidate to be used for the treatment of AD. In addition to this, ENPs can affect the native protein stability, alter the rate of folding and unfolding processes by acting as chaperones [100,116].

### 5.3. Nanozymes

Advances in ENPs synthesis have led to the development of some inorganic ENPs (such as ceria nanoparticles and iron oxide nanoparticles) with enzyme-mimetic ability. These ENPs (also known as nanozymes) as compared to natural enzymes have more robust catalytic activities, can work at temperature and pH conditions of a wide range, have cost-effective production and greater flexibility in designing. These properties make nanozymes a good candidate for future medicines [117]. 

### 5.4. ENPs for Drug Delivery

ENPs can prove to be a promising strategy for drug delivery for neurodegenerative and conformational diseases. Owing to their binding with the external region of amyloid-β fibrils (which are known to play a crucial role in fibrillation), monolayer-protected ENPs like AuNPs can be applied for preventing secondary nucleation, fibril aggregation and as drug delivery agents to specified fibril regions [118]. Chaperone mimicking ENPs made up of polymeric micelles which can mimic Hsp70 were found to be a good method for the protection and delivery of insulin in hyperglycaemic patients [119]. 

These studies showed that ENPs can be used as potential drugs, drug carriers and can complement currently used therapeutic strategies for protein misfolding diseases and neurodegenerative disorders (Table 3).

**Table 3:** Studies showing involvement of ENPs in protein modulation.

ENPs	Protein/Model Used	Disease	Outcome	Reference
Zinc oxide nanoparticles (ZnO NPs), short ZnO nanorods (s-ZnO NRs), and long ZnO nanorods (l-ZnO NRs)	Human and zebrafish larvae neuroblastoma cells SH-SY5Y	Parkinson’s disease (PD)	PD like symptoms developed	[120]
CuO nanoparticles (CuONP), Fe_2_O_3_ nanoparticles (Fe_2_O_3_NP), ZnONP	Rat cell lines (PC12) and human SH-SY5Y and H4 cells	Alzheimer’s disease (AD)	Concentration-based neurotoxicity of CuONP but not Fe_2_O_3_NP and ZnONP. CuONP as an environmental risk factor for AD	[121]
Poly(trehalose) nanoparticles	HD150Q cells, HD transgenic mice [B6CBA-Tg (HDexon1) 62Gpb/3Jstrain]	Huntington’s disease (HD)	Inhibition of amyloid aggregation and prevention of polyglutamine aggregation.	[122]
Carbon nanoparticles (graphene and carbon nanotubes)	Mouse prion protein (moPrP117−231)	Prions disease	Carbon nanoparticles inhibited Prion fibrillation in In vitro studies SWCNT and graphene reduced interaction of peptide and caused the formation of β-structure	[123]
Nanoliposomes (NL)	Purified AL light chain proteins, ex-vivo human arteriole model, Human aortic artery endothelial cells (HAEC)	Light chain amyloidosis (AL)	Increased folded protein amount, reduced cell internalization.	[80]
Dendrimer–tesaglitazar	BV2 murine microglial cell line	AD and PD	Microglial phynotype shift, increased β-amyloid phagocytosis	[114]
Graphene QDs	10 DIV mouse cortical neurons, C57BL/6 mice	PD	Inhibition of α-syn fibril formation, trigger fibril disaggregation, protects against dopaminergic neuron loss and Lewy body pathology	[124]
N-methyl D-aspartic acid functionalized gold nanoparticle (GNP-NMDA)	Low molecular weight (LMW) amyloid oligomers	AD	Inhibition of LMW tetramer of amyloid oligomer towards nontoxic aggregation path	[125]
Protein capped Fe_3_O_4_ (PC-Fe_3_O_4_) and PC-CdS	Tau protein	AD	Inhibition of Tau aggregation	[126]

## 6. Techniques to Study ENP-Protein Interactions

ENP-protein system studies are focused on investigating alterations occurring in both the components of the system and in combination. The information generated from such studies can reveal the structural, geometrical, topographical, and physicochemical details about the ENP-protein system. Based on the purpose of the study, there are numerous characterization techniques available for analyzing ENP-protein interactions [127,128,129]. The techniques used to study the ENP-protein system can be categorized as spectroscopic, microscopic, thermodynamic, separation and in silico techniques (Table 4).

**Table 4:** ENP-protein interaction studies done using various techniques.

Techniques Applied	ENPs	Proteins	Parameters Analyzed	References
FTIR spectroscopy, UV–vis spectrophotometry, TEM, fluorescence and, CD spectroscopy	AuNPs	Bovine serum albumin (BSA)	Amount of α-helical structure Conformational change in proteins, secondary and tertiary structural alterations in proteins	[130,131]
DLS, TEM, Far-UV spectra, CD spectroscopy	Unmodified TiO_2_	α-chymotrypsin, RNase A, and papain	Protein refolding	[132]
UV/vis spectrophotometry, Raman spectroscopy, electronic paramagnetic resonance (EPR) spectroscopy	Two amorphous pyrogenic silica ENPs	Bovine serum albumin (BSA), hen egg lysozyme (HEL), bovine pancreatic ribonuclease A, RNase and bovine lactoperoxidase (LPO)	Quantify adsorbed protein, surface-driven structural modification, protein orientation on the nanoparticle surface	[129]
SDS-PAGE, densitometry, AFM, analytical ultracentrifugation (AUC)	SiO_2_ and CeO_2_ nanoparticles	Serum proteins and BSA	Adsorption behaviour of proteins	[133]
Affinity chromatography, UV-visible spectroscopy, TEM, SANS, CD	Silica nanoparticles	Green fluorescent protein (GFP)	Relationship between unfolded proteins, silica nanoparticles and chaperonin	[134]
TEM, CD	Ultra-small AuNPs	Amyloid β	Inhibition of fibrillation process, disruption of peptide folding process	[135]
MD simulation	Monolayer-capped AuNPs	Amyloid β fibrils	Location and binding affinity of nanoparticles with proteins	[118]
ThT, Congo red assay, FTIR, CD, AFM	Silica nanoparticles (SiNPs)	Hen egg-white lysozyme (HEWL)	Aggregation behavior	[136]

### 6.1. Spectroscopic Techniques 

There are numerous spectroscopic techniques available for the characterization of ENPs-protein systems like UV-visible spectroscopy, circular dichroism (CD) spectroscopy, Fourier-transform infrared (FTIR) spectroscopy, Raman spectroscopy, nuclear magnetic resonance (NMR), mass spectrometer, dynamic light scattering (DLS), Bradford assay, etc. which are used for characterization, quantification, the study of structural transitions and chemical bonding present in ENP-protein system [54,56,137]. These techniques provide data in the form of spectral peaks which are then analyzed to obtain desired information. CD spectroscopy shows spectral peaks for secondary structures at different wavelengths as α- helices show negative bands at 208 nm, 222 nm and a positive band at 193 nm, β-pleated sheets have a negative band at 193 nm and a positive band at 195 nm and disordered proteins show very low ellipticity above 210 nm and a negative band at 195 nm [138]. CD spectroscopy is used to analyze the extent of structural (α-helix, β-sheets, disordered structures) changes occurring in protein due to interaction with ENPs which are indicated by changes in the spectral peaks. Fourier-transform infrared spectroscopy (FTIR) is another widely used technique that provides details of secondary structural changes like α-helix, β-sheets, β-turns and irregular structures based on the change in the vibrational frequency of amide modes (amide I due to C=O stretching vibration, amide II which is primarily due to N-H bending and some from C-N stretching vibrations and amide III due to N-H bending and C-N stretching vibrations). Out of these, the amide I band which is in the range of 1600–1700 cm^−1^ is the most commonly studied region for secondary structural analysis [139]. CD spectroscopy and FTIR spectroscopy techniques have been widely used for analyzing secondary structural changes in proteins on ENPs exposure [109,140]. Raman spectroscopy is another important technique that is similar to IR spectroscopy with higher sensitivity and can be used for examining the deformation extent of proteins on interaction with ENPs [129]. Electron paramagnetic resonance (EPR) spectroscopy with spin-labeled proteins is used to study the protein orientation on the surface of the nanoparticles. 

Scattering techniques are used for investigating structural and interactional aspects of the ENPs-protein system. Probing of ENP-protein interactions are done using dynamic light scattering (DLS), small-angle X-ray scattering (SAXS) and small-angle neutron scattering (SANS) techniques. In DLS, particle diffusion coefficients provide information about the structural and interactional aspects, while in SANS and SAXS these two contributions can be separated. SANS and SAXS can be used for the determination of structural and thermodynamic aspects of ENPs−protein complexes under physiological conditions [141].

In addition to these, there are various assays for qualitative and quantitative analysis of proteins exposed to ENPs. These assays are based on absorbance, fluorescence and luminescence. Absorbance-based assays such as Bicinchoninic acid (BCA) assay and Bradford assay are known methods for quantification of the amount of adsorbed proteins on ENPs. BCA assay works at λ562 nm and Bradford assay at λ595 nm. Thioflavin T (ThT) assay is a fluorescence-based assay that has been extensively applied for studying protein misfolding and aggregation when exposed to ENPs [115]. This dye binds specifically to β sheets (linked to protein misfolding) of proteins. The enzyme activity assays have been used to study the functioning of proteins exposed to ENPs [142]. Since protein function is directly linked to the correct structure and folding of the proteins, therefore enzyme activity assays can provide information about the stability of native proteins and the extent of refolding of unfolded proteins in presence of ENPs. 

### 6.2. Microscopic Techniques

These are advanced techniques that have revolutionized the area of ENP-protein interaction studies. Direct visualization of the ENP-protein system is done using microscopic techniques like optical microscopy, electron microscopy techniques (like transmission electron microscopy (TEM), scanning electron microscopy (SEM)) and atomic force microscopy (AFM). TEM is an important electron microscopy-based technique that can be used for morphological study of ENPs, visualizing protein corona and for cross-checking of newly developed techniques such as pre-adsorption strategy for targeting moieties that are attached to the surface of nanocarriers [143]. Cryo-electron microscopy (cryo-EM) is an advanced electron microscopy technique using which proteins can be observed without staining. Visualization and discrimination of hard and soft corona can be done using TEM and cryo-EM [144]. SEM is applied for visualizing changes in ENPs morphology due to the binding of proteins onto the ENP surface [145]. Atomic force microscopy (AFM) is used to analyze topology and it is used for visualizing fibrillation and aggregation of proteins exposed to ENPs [111].

### 6.3. Thermodynamic Techniques

Thermodynamic parameters like free energy and entropy of the ENPs-protein system are analyzed for their crucial role in deciding the spontaneity and possibility of ENPs-protein interactions. Techniques such as differential scanning calorimetry (DSC) and isothermal titration calorimetry (ITC) have been used for thermodynamic analysis of ENPs-protein interactions, comparing binding affinity of different ENPs for proteins and spontaneity of the binding process [95]. DSC is applied to calculate the change in free energy of the thermal denaturation of proteins. Therefore, this technique provides information about the stability of proteins after it is adsorbed on ENPs surface. ITC is an important technique that is applied for the measurement of affinity, free energy and stoichiometry of ENP-protein interaction. The negative free energy (ΔG) is an indicator of spontaneous binding between protein and ENPs. 

### 6.4. Separation Techniques

These techniques are used to investigate the identities of adsorbed proteins and provide information about the protein coronas. Various types of gel electrophoresis techniques include sodium dodecyl sulfate-polyacrylamide gel electrophoresis (SDS-PAGE), difference gel electrophoresis (DIGE), PAGE (1D or 2D), capillary electrophoresis (CE) and agarose gel electrophoresis. To investigate adsorbed protein identities, SDS-PAGE is used. These can be categorized as one-dimensional (1-DE) gel electrophoresis and two-dimensional (2-DE) gel electrophoresis. In 1-DE, proteins present in the protein mixture are separated based on their molecular weight. In 2-DE, protein separation is done in two steps, firstly isoelectric focusing (IEF) is performed, where proteins get separated according to their isoelectric points (IEP), and secondly SDS-PAGE which is used to separate proteins based on their molecular weights [127]. CE is an important separation technique with the advantage of high-resolution power, speed, efficiency and facilitating quantification of adsorbed proteins on ENPs surface without undergoing desorption process. It is used to identify ENP-protein coronas, separation, and characterization of individual ENPs that are in conjugation with proteins [146].

Chromatography techniques such as mass spectrometry (MS) are used for the identification of proteins forming protein corona. To investigate the affinity of proteins towards ENPs, the size-exclusion chromatography technique is used [147]. Electrophoresis coupled with MS is the most efficient and widely used combination of methods for separating proteins and complex protein mixtures analysis [148].

### 6.5. In Silico Techniques

Computational biology-based techniques for ENP-protein interaction studies play an essential role in understanding the possible alterations caused due to interaction between ENPs and proteins. These techniques helped to predict the binding sites of ENPs on proteins, conformational changes of proteins, preference of interaction with different amino acids. ENP-based drug designing for drug delivery is also done using in silico approaches [149,150,151]. These studies use online databases available for proteins such as Protein Data Bank (PDB), Swiss-Prot. Common techniques applied for such studies include molecular docking, molecular dynamics simulations (MD simulations), Replica Exchange Solute Tempering Molecular Simulations (REMD) and Replica Exchange with Solute Tempering (REST) [149,152,153]. REST was used to understand the importance of electrostatic interactions in driving protein (such as amyloid-β monomers) adsorption over ENPs (like citrates-coated AuNPs) [153]. 

## 7. Discussion

Considering the extensive use of ENPs in various consumer and industrial products, there is an emerging concern about the effects of ENPs on living organisms. Owing to their nano size, shape, surface charge and other physicochemical properties, ENPs can penetrate cells, cross the blood-brain barrier (BBB), interfere with the normal functioning of various cellular components, and interact with biomolecules. Recently, the interaction of ENPs with proteins has received a lot of attention because of the inevitable role of proteins in different life processes and the tendency of ENPs to interact with proteins. Several studies have been done till now to understand the possible consequences of ENP-protein interactions. These studies confirm that ENPs and proteins mutually alter each other, changing the bio reactivity of ENPs and causing alterations in protein conformation and functional efficiency. ENP-protein interactions depend on various factors such as shape, size, surface charge, hydrophobicity, hydrophilicity, chirality of ENPs and orientation, free energy, binding affinity, size of proteins. The anti-amyloid properties of ENPs which help to prevent and inhibit the amyloidosis process (linked to various protein misfolding and neurodegenerative diseases) make it more important to understand the pathways involved in ENPs- protein interactions. 

In natural systems, molecular chaperones maintain protein homeostasis and failure of chaperones machinery may result in diseased conditions. Therefore, ENPs which can mimic molecular chaperones can complement existing treatment strategies available for protein misfolding diseases. These ENPs assist the folding of proteins by unfolding misfolded proteins and further refolding them to their native form, inhibiting biologically deleterious processes such as protein misfolding, aggregation and fibrillation. Therefore, this necessitates the study of ENP-protein interactions to a deeper level as the knowledge generated from these studies can help in understanding the causes, mechanisms, consequences of these interactions and develop ENPs based drugs for conformational diseases which are considered incurable till now.
